# Metabolomics profiling reveals berberine-inhibited inflammatory response in human gingival fibroblasts by regulating the LPS-induced apoptosis signaling pathway

**DOI:** 10.3389/fphar.2022.940224

**Published:** 2022-08-22

**Authors:** Ying Zhang, Yanyang Guo, Wenjia Wei, Zhongxiao Zhang, Xiaodong Xu

**Affiliations:** ^1^ Department of Stomatology, Tongren Hospital, Shanghai Jiaotong University School of Medicine, Shanghai, China; ^2^ Hongqiao International Institute of Medicine, Tongren Hospital, Shanghai Jiao Tong University School of Medicine, Shanghai, China

**Keywords:** berberine, inflammatory, human gingival fibroblasts, periodontitis, metabolomics

## Abstract

This article examines berberine’s biological effects and molecular mechanisms with an inflammatory response model induced by lipopolysaccharide (LPS) in human gingival fibroblasts (HGFs) using metabolomics. The viability of HGFs was determined using the cell counting kit-8 (CCK8). ELISA was used to measure inflammatory cytokines, including interleukin-6 (IL-6), interleukin-1*β* (IL-1*β*), and tumor necrosis factor- *α* (TNF-*α*). An investigation of western blots was conducted to investigate the related proteins of apoptosis. Low concentrations of berberine (0.1, 0.5, and 1 μmol L^−1^) did not affect HGF growth, whereas high concentrations of berberine (5–25 μmol L^−1^) significantly activated cell proliferation. Berberine suppressed the elevated secretion of IL-6, IL-1β, and TNF-α induced by LPS in HGF. Western blot analysis showed that 10 μmol L^−1^ of berberine significantly inhibited LPS-induced apoptosis signaling pathway activation. Our results suggested that berberine could inhibit LPS-induced apoptosis and the production of proinflammatory mediators in HGFs cells. Berberine may be a potential therapeutic drug for the management of periodontitis.

## Introduction

Humans are affected by periodontitis, one of the most common oral diseases. It has a high prevalence worldwide and is the first cause of tooth loss in adults (Hajishengallis and Chavakis, 2021). At the same time, periodontitis can also cause oral and maxillofacial inflammation and many systemic diseases (Kapila, 2021), such as cardiovascular disease (Lăzureanu et al., 2021), diabetes (Casillas Santana et al., 2021), and premature weight loss. With the increasingly serious problem of the aging society in the world, the social demand for oral restoration will be greater to prevent periodontal disease.

Berberine hydrochloride, also known as berberine, is an isoquinoline alkaloid derived from the skin and root of *Coptis chinensis* in Ranunculaceae (Song et al., 2020). Early experiments have proven that berberine hydrochloride has obvious pharmacological effects on pathogenic microorganisms (Wang et al., 2017). In recent years, it has been found that berberine exerted various pharmacological effects such as hypoglycemic, anti-tumor, anti-arrhythmia, anti-inflammatory, and immune regulation (Ren et al., 2021). Clinical research and animal experiments have confirmed that the drug has a good therapeutic effect, can improve the condition of the whole body and periodontal tissue, increases alveolar bone density, and reduces the inflammation and destruction of periodontal tissue (Chen et al., 2018). However, the mechanism of berberine hydrochloride in the treatment of periodontal disease is not very clear.

In humans, gingival fibroblasts are the main cellular type that composes the soft periodontal tissues and triggers immune responses by secreting cytokines and chemokines. Consequently, HGFs play a major role in host immunity and inflammation (Mohammadian Haftcheshmeh and Momtazi-Borojeni, 2021). For example, HGFs produce proinflammatory cytokines, interleukin-6 (IL-6) and interleukin-8 (IL-8), which are closely linked to periodontitis and are associated with periodontal tissue destruction and alveolar bone loss (Zhang et al., 2019). Due to the importance of HGFs in maintaining the integrity of periodontal tissue and the regulation of periodontal inflammation (Naruishi and Nagata, 2018), it is of great significance to find active substances targeting HGFs in the prevention and remedy of periodontitis. At the same time, in view of the anti-inflammatory effect of berberine, it is necessary to study the biological function of berberine on HGFs.

With advances in high-throughput analytical techniques for metabolomics research, studies have shown metabolic signatures in disease diagnosis (Nakashyan et al., 2017), pathogenesis (Kang et al., 2018), preclinical safety evaluation (Lee et al., 2019), and drug discovery and development (Rinschen et al., 2019). However, the metabolic profile during the period of the treatment of periodontitis has not been defined (Wishart, 2019). Berberine has been shown to improve glycolipid metabolism at present (Guleria et al., 2018). Additionally, apical periodontitis has been associated with metabolic disorders (Yeung, 2018), and missing teeth (or edentulousness), which is the endpoint of periodontitis and caries, has been shown to be strongly associated with metabolic disorders (Baima et al., 2021). It is of great significance to study whether berberine can reduce periodontitis by reversing metabolism disorder. However, the metabolic regulation of berberine on other inflammatory diseases has been studied. There is still no metabolomics study on the effect of berberine on the treatment of periodontitis with the HGFs inflammatory model. Therefore, it is urgently necessary to devise a more accurate, efficient, and convenient method for monitoring dynamic changes in the process of periodontitis treatment with berberine, exploring the pharmacological mechanisms of berberine in the treatment of periodontitis.

In the present study, an inflammatory model induced by LPS in HGFs was developed to explore the inflammatory and metabolic regulation effect of berberine. We aim to explore the mechanism of berberine and establish a theoretical foundation for the better use of natural drugs in the treatment of periodontitis.

## Methods

### Reagents

Berberine hydrochloride and LPS were obtained from Sigma Chemical Co. (St. Louis, Missouri, United States). We purchased mouse ELISA kits that detect TNF-*α*, IL-6, and IL-1*β* from Shanghai Chuanfu Biotechnology Co., Ltd. We purchased acetate (HPLC grade) and methanol (HPLC grade) from Fisher (United States). Milli Q system (Millipore, Bedford, MA, United States) was used to obtain a very pure water sample. Human gingival fibroblasts (HGFs) have been isolated by Sciencell Research Laboratories using human gingival tissue as a source.

### Cultivation*,* apoptosis, and cell-viability analysis

There were three groups: a normal control group (NC), a lipopolysaccharide (LPS) group with a vehicle, and LPS + Ber group. HGFs were cultivated in a fibroblast medium supplemented with penicillin (100 U/ml), streptomycin (100 U/ml), 2 mmol/L glutamine, and 10% fetal bovine serum (FBS) (HyClone, Logan, Utah, United States) at 37°C with 5% CO_2_ (normalized conditions). The LPS + Ber group cells were seeded in a 96-well plate and treated with berberine (0, 0.1, 0,5, 1, 2.5, 5, 10, 25, 50, and 100 μM) for 24, 48, and 72 h and stimulated with LPS (100 ng/ml) for 24 h. A total of 20 μL CCK8 (5 mg/ml) was added to each well for an additional 4 h. We measured TNF-*α*, IL-1*β*, and IL-6 levels using the ELISA kits according to the protocol.

### Western blot analysis

For total protein collection, cells were washed in PBS and lysed with RIPA lysis. Bradford Assay Reagent kit was used to determine the protein concentration. Protein samples of the same amount (40 μg/lane) were subjected to SDS-PAGE (10% tricine gels). After that, proteins were transferred onto PVDF membranes. After blocking with 5% BSA at room temperature for 1 h, the membranes were incubated with primary antibodies against BAX, PARP, Caspase-9, Caspase-3, Cytochrome C, and GAPDH (1:1,000, Cell Signaling Technology) at 4°C overnight. Afterward, a secondary antibody was incubated with the membranes at 25°C for an hour. Standardization was performed using an antibody against GAPDH. The detection of the immunoreactive band was done by Supersignal West PicoLuminol (Thermo, Waltham, MA, United States), and Image J software was used for quantification.

### Detection of apoptosis of HGFs

Flow cytometry analysis was used to assess apoptosis of the HGFs after staining with the annexin V-FITC/PI apoptosis detection kit. Briefly, cells were gently resuspended in annexin V binding buffer and then incubated with annexin V-FITC/PI for 15 min in the dark. After the cells had been fixed, they were washed twice with PBS and stained with 20 g/ml propidium iodide (PI), and samples were analyzed by flow cytometer. Each sample was collected from at least 10,000 events. Annexin-Vneg/PIneg (unlabeled) is the marker for viable cells, annexin-Vneg/PIpos is for necrotic cells, and annexin-Vpos and PI-neg and PI-pos are for early and late apoptotic cells, respectively.

### Processing for metabolomic analysis

HGF cells were washed twice with 500 µL of ice-cold methanol containing internal standards (25 mM each of 2-chlorophenylalanine). The methanol extract (supernatant) was collected, and the mixture was centrifuged at 10,000 rpm for 10 min at 4°C. The centrifuged supernatant was obtained and stored at −80°C for metabolic analysis. All serum samples with the same volume are mixed for quality control (QC).

### UPLC-ESI-MS/MS analysis

Non-targeted metabolomics analysis was performed using Ultimate 3000 UPLC (Dionex, Sunnyvale, CA, United States) tandem orbitrap^TM^ mass spectrometer (Thermo, MA, United States) with an electrospray ion source. Data-dependent (dd-MS2, TopN = 20) MS/MS mode with a full scan mass resolution of 35,000 at an m/z of 200 was used. The scanning range was m/z 50–1500, and positive and negative ions were collected separately with an MS/MS resolution of 17,500. Collision energy 35ev; automatic gain control (AGC) target was 5e5. A reversed-phase ACQUITY UPLC HSS T3 column (100 Å, 1.8 μm, 2.1 × 100 mm) was used for chromatographic separation. The chromatographic conditions were set as follows: flow rate 0.35 ml/min, injection volume 3 ul, and column temperature 35°C. In phase A, water was combined with 0.1% formic acid, whereas in phase B, 0.1% formic acid was combined with acetonitrile. The mobile phase gradient is 0–2 min 5% B, 2–10 min 5%–95% B, 10–15 min 95% B, and 15–18 min 95%–5% B, and the total running time was 18 min. Data were acquired in centroid mode using Thermo Excalibur 3.2 software (Thermo Scientific, MA, United States).

### Data analysis for metabolomics

Data files from metabolomics were analyzed by compound discoverer 3.2 (Thermo Fisher Scientific). Data was filtered, identified peaks were matched across samples, and the missing peak data were filled in. The corresponding parameter settings in Compound Discoverer 3.2 were as follows: the minimum intensity threshold of the peak response was set to 10,000, the mass tolerance to 10 ppm, and the retention time (RT) tolerance to 0.4 min. Use mzVault, Chemspider, and a self-built standard database for metabolite identification.

Data were exported into SIMCA-P+ 13.0 software (Umetrics AB, Umeå, Sweden) for PCA and PLS-DA analysis. Metabolite peaks were identified by MSE analysis and annotated with available biochemical online databases, METLIN, HMDB, Lipid Maps, and Chemspider. Student’s *t*-test was used to screen statistically significant metabolites when data distribution followed the normality assumption. In other cases, Mann–Whitney *U* tests were used. Significantly changed metabolites were those with a *p*-value < 0.05 and a VIP greater than 1. Metabolic pathway analysis was performed by MetaboAnalyst 3.0 using differential metabolites. The regulated metabolites were imported into IPA software for network construction.

## Results

### Differential metabolite screening

**FIGURE 1:**
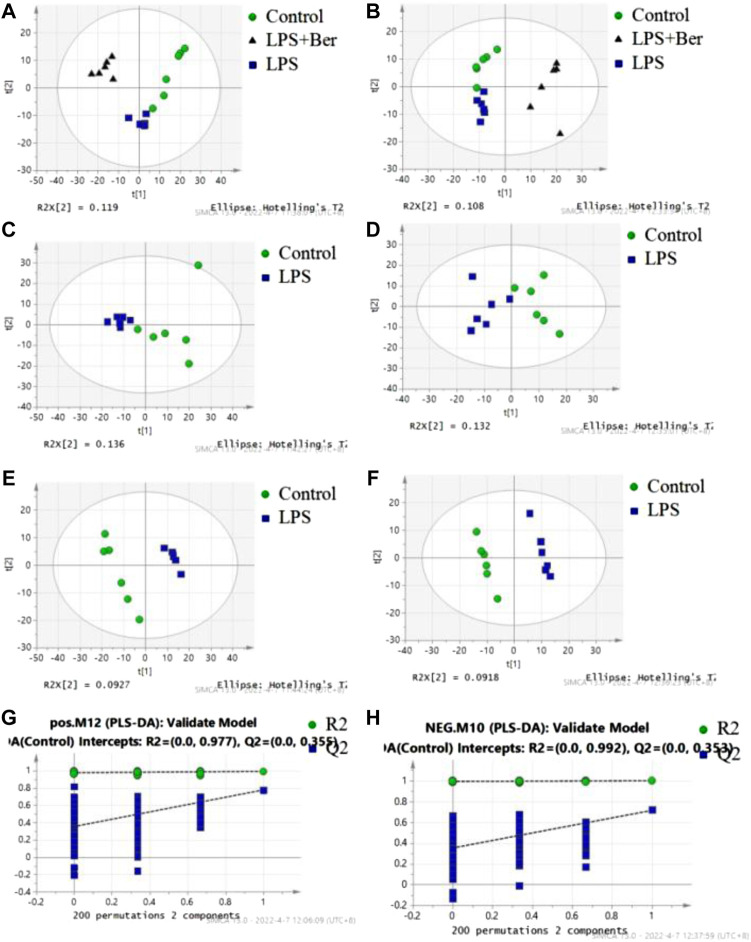
PCA and PLS-DA analysis of metabolites in the normal control (NC), LPS, and LPS + Ber groups. **(A,C,E,G)** Positive ion mode. **(B,D,F,H)** Negative ion mode. **(A,B)** PCA score plots discriminating the LPS, NC, and LPS + Ber groups in positive and negative ion modes. **(C,D)** PCA score plots discriminating the LPS and NC groups. **(E,F)** PLS-DA score plot of the LPS and NC groups. **(G,H)** Permutation test of PLS-DA model. ●NC group; ▲LPS + Ber.▪ LPS.

**FIGURE 2:**
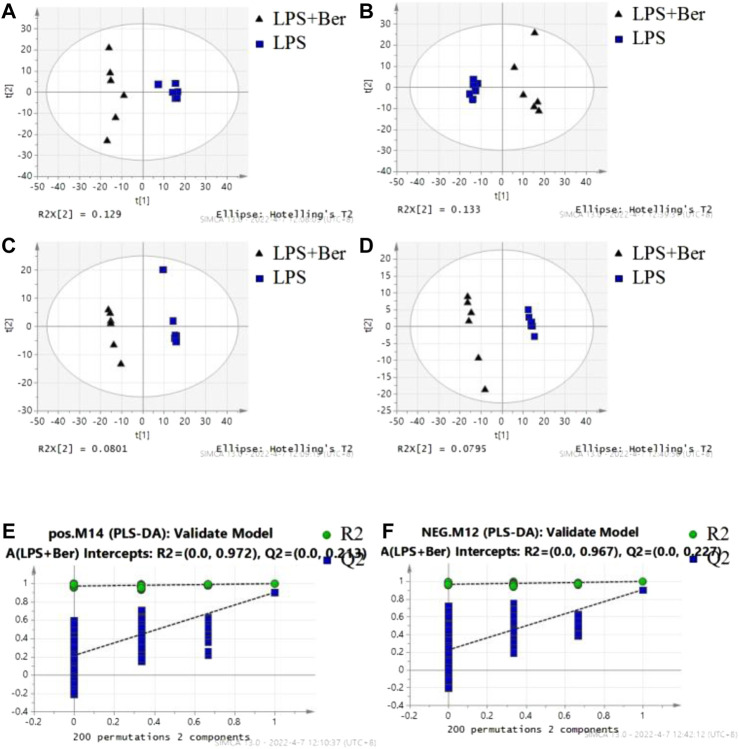
The results of multiple pattern recognition of metabolites between LPS and LPS + Ber group. **(A)** PCA score plot under positive ion mode. **(B)** PCA score plot under negative ion mode. **(C)** PLS-DA score plot under positive ion mode. **(D)** PLS-DA score plot under negative ion mode. **(E,F)** Permutation test of PLS-DA model.

**FIGURE 3:**
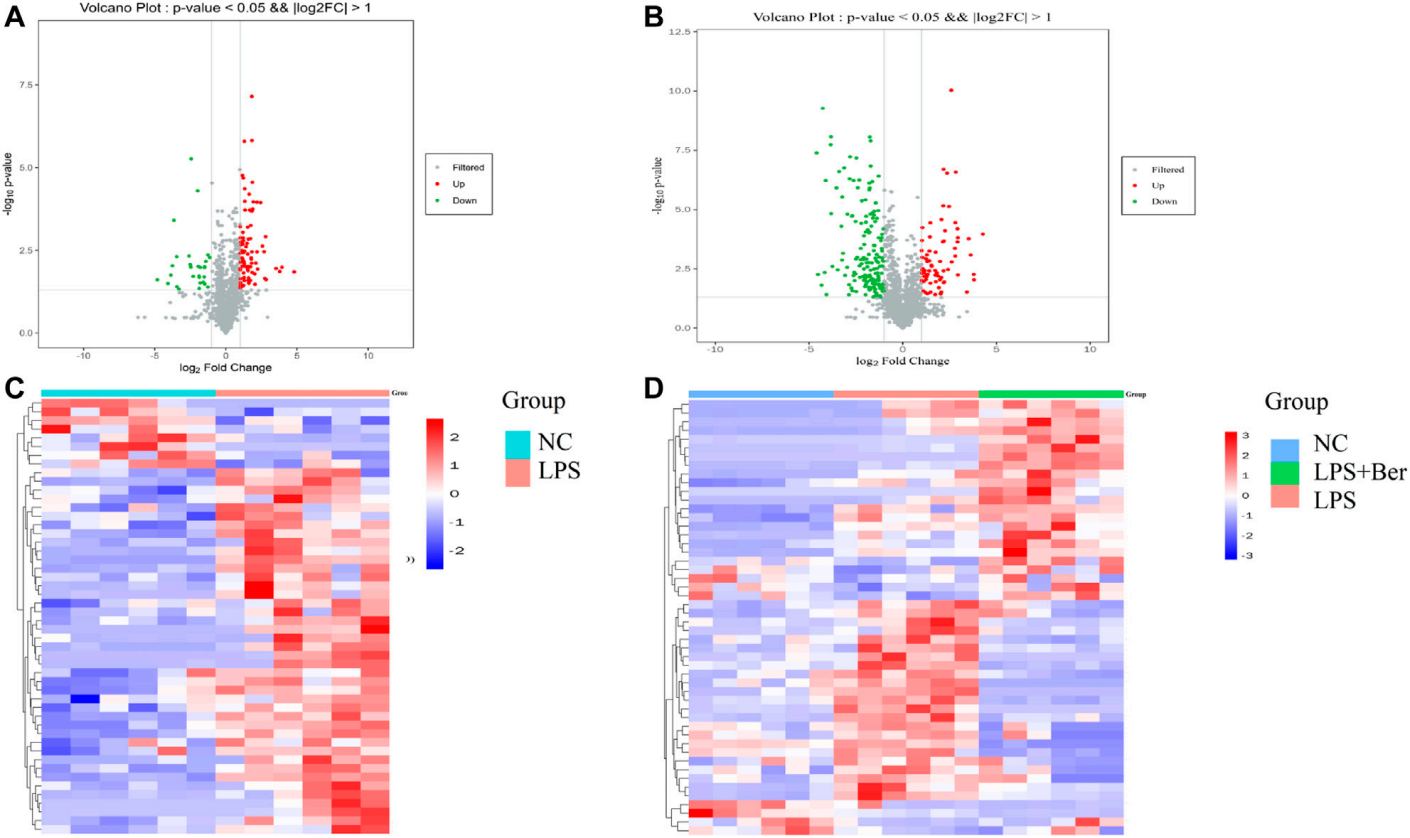
**(A)** Volcano plot of LPS *versus* NC. Red circles have an FDR value <0.05, indicating a significant increase in LPS. Green circles have an FDR value <0.05, indicating a significant decrease in LPS. **(B)** Volcano plot of LPS *versus* LPS + Ber. Red circles have an FDR value <0.05, indicating a significant increase in LPS. Blue circles have an FDR value <0.05, indicating a significant decrease in LPS.**(C)** A heatmap shows that the top 20 metabolites can separate LPS and NC. **(D)** A heatmap shows that the top 20 metabolites can separate LPS, NC, and LPS + Ber groups.

There were 900 peaks detected in the ESI+ mode and 747 peaks in the ESI− mode. The PCA and PLS-DA were performed to show an overview of LC-MS data collected from the serum of different groups. The PCA score plots (Figure 1) showed an obvious separation between the control group, the LPS group, and LPS + Ber group, both in ESI+ and ESI−. In addition, the LPS + Ber group was close to the normal group. The comparison between the LPS group and LPS + Ber was also analyzed by PCA and PLS-DA. As shown in Figure 2, the Ber can change the metabolic profile of the LPS group. The volcano plot and heatmap were used to show differential metabolites between the LPS *versus* control group and the LPS *versus* LPS + Ber group, as shown in Figure 3. A total of 52 metabolites with statistical significance (VIP > 1, *p* < 0.05) were identified and listed (Table 1). In brief, the levels of PC (16:1/22:6), PE (16:0/18:1), PC (18:2/22:6), PE-NMe (24:0/24:1(15Z)), PE (18:1/18:1), PE-NMe (18:1/18:1), and PS (O-16:0/21:0) were significantly increased in LPS group, whereas the levels of PS (24:0/24:1), TG (20:2/20:4/20:4), TG (20:1/20:1/20:4), PS (O-18:0/19:1), PS (15:0/20:1), and LysoPC (16:1/0:0) were significantly decreased in the LPS group than those in the normal control group (*p* < 0.05). However, berberine might alleviate the metabolic perturbation in HGFs caused by LPS.

**TABLE 1:** 52 serum metabolites significantly changed in the comparison of the LPS/NC and LPS + Ber/LPS.

Molecular weight	RT (min)	Name	HMDB	VIP (LPS/C)	*p* (LPS/NC)	log2FC (LPS/C)	log2FC (LPS + Ber/LPS)
957.74706	15.31	PS(24:0/24:1(15Z))	NA	1.65	4.71E-03	−2.59	0.02
954.75078	9.99	TG(20:2(11Z,14Z)/20:4(5Z,8Z,11Z,14Z)/20:4(5Z,8Z,11Z,14Z))	NA	1.48	2.92E-02	−1.59	1.18
962.8333	16.84	TG(20:1(11Z)/20:1(11Z)/20:4(5Z,8Z,11Z,14Z))	HMDB0005465	1.33	3.29E-02	−1.49	0.00
789.59419	12.26	PS(O-18:0/19:1(9Z))	NA	1.95	1.90E-03	−0.87	0.15
775.53784	9.57	PS(15:0/20:1(11Z))	NA	1.61	2.41E-02	−0.81	−0.34
493.31722	7.55	LysoPC(16:1(9Z)/0:0)	HMDB0010383	1.69	7.82E-03	−0.79	0.31
763.57868	16.91	PS(O-18:0/17:0)	NA	1.57	2.67E-02	−0.61	1.31
805.5939	7.17	PE(O-20:0/22:6(4Z,7Z,10Z,13Z,16Z,19Z))	NA	1.59	2.19E-02	−0.57	0.82
831.60798	13.75	PS(15:0/24:1(15Z))	NA	1.45	4.31E-02	−0.44	0.67
849.54749	16.98	PS(19:0/22:6(4Z,7Z,10Z,13Z,16Z,19Z))	NA	1.33	3.99E-02	0.03	2.83
741.56407	11.96	PC(16:1(9Z)/P-18:1(11Z))	HMDB0008029	1.44	1.66E-02	0.04	2.21
763.54616	8.07	PC(18:4(6Z,9Z,12Z,15Z)/P-18:1(11Z))	HMDB0008260	1.49	4.59E-02	0.23	−2.48
807.57594	16.98	PC(16:0/22:5(4Z,7Z,10Z,13Z,16Z))	HMDB0007989	1.31	3.61E-02	0.36	2.80
862.56832	17.12	PI(16:0/20:2(11Z,14Z))	HMDB0009786	1.50	3.37E-02	0.42	−0.62
165.07914	2.40	L-Phenylalanine	HMDB0000159	1.45	2.12E-02	0.42	−0.10
136.03866	0.89	Hypoxanthine	HMDB0000157	1.48	3.62E-02	0.50	−1.80
537.5117	13.85	N-Palmitoyl-D-erythro-sphingosine	NA	1.27	4.49E-02	0.64	−0.36
811.54759	8.11	PS(16:0/22:4(7Z,10Z,13Z,16Z))	NA	2.14	1.71E-04	0.70	−0.47
743.51994	11.26	PS(O-16:0/18:3(9Z,12Z,15Z))	NA	1.97	1.48E-03	0.81	−0.77
934.70043	16.95	TG(17:2(9Z,12Z)/20:5(5Z,8Z,11Z,14Z,17Z)/22:6(4Z,7Z,10Z,13Z,16Z,19Z))	NA	1.33	3.20E-02	0.82	−1.15
777.56223	8.35	PE(22:4(7Z,10Z,13Z,16Z)/P-18:1(11Z))	HMDB0009611	1.76	8.14E-03	0.84	−1.89
717.52803	13.96	PE(18:1(9Z)/16:0)	HMDB0009055	1.43	4.78E-02	0.84	−0.44
765.5309	16.99	PE-NMe(15:0/22:5(7Z,10Z,13Z,16Z,19Z))	NA	1.48	1.57E-02	0.85	−0.58
765.56628	11.96	Eicosapentaenoyl PAF C-16	NA	1.34	2.89E-02	0.94	1.14
715.51682	17.00	PE(16:1(9Z)/18:1(11Z))	HMDB0008959	1.68	2.99E-03	0.96	−1.54
805.56072	13.52	PC(16:0/22:6(4Z,7Z,10Z,13Z,16Z,19Z))	HMDB0007991	1.52	1.25E-02	1.01	0.78
854.73584	16.54	TG(17:2(9Z,12Z)/17:2(9Z,12Z)/18:0)	NA	1.60	5.41E-03	1.14	−3.23
789.52963	17.00	PE(18:1(11Z)/22:6(4Z,7Z,10Z,13Z,16Z,19Z))	HMDB0009045	1.66	3.22E-03	1.15	−0.45
755.54225	11.19	PC(14:0/20:3(5Z,8Z,11Z))	HMDB0007881	1.59	5.82E-03	1.18	2.18
831.57527	11.99	PC(18:1(11Z)/22:6(4Z,7Z,10Z,13Z,16Z,19Z))	HMDB0008090	1.56	7.16E-03	1.19	0.59
833.61519	8.45	PS(15:0/24:0)	NA	1.49	3.75E-02	1.19	−0.74
743.54249	7.57	PE(18:1(9Z)/18:1(9Z))	NA	1.48	3.65E-02	1.24	−0.34
747.5273	13.06	PE(P-16:0/22:6(4Z,7Z,10Z,13Z,16Z,19Z))	HMDB0005780	1.77	7.79E-03	1.29	−0.45
607.07893	1.17	Uridine diphosphate-N-acetylglucosamine	HMDB0000290	2.22	4.33E-05	1.33	−2.40
765.52691	13.91	PC(15:0/20:5(5Z,8Z,11Z,14Z,17Z))	HMDB0007951	1.73	9.80E-03	1.40	−1.08
767.54688	16.99	PE-NMe(15:0/22:4(7Z,10Z,13Z,16Z))	NA	1.71	1.96E-03	1.49	0.40
773.53593	11.31	PE(22:6(4Z,7Z,10Z,13Z,16Z,19Z)/P-18:1(11Z))	HMDB0009710	1.75	1.45E-03	1.58	0.27
761.52205	7.99	PS(16:0/18:1(9Z))	HMDB0012357	1.52	3.11E-02	1.62	1.00
777.55607	6.84	PS(15:0/20:0)	NA	2.15	2.03E-04	1.76	0.19
648.50954	16.63	PA(O-18:0/15:0)	NA	2.11	7.11E-08	1.82	0.37
913.77554	16.56	PE-NMe2(22:0/24:1(15Z))	NA	1.65	3.52E-03	1.83	−1.22
739.54991	16.97	PC(18:3(6Z,9Z,12Z)/P-16:0)	HMDB0008192	2.08	1.54E-06	1.83	0.61
185.99326	1.18	2-Phosphoglyceric acid	HMDB0000362	1.93	2.05E-04	1.84	−2.07
723.51716	13.96	PE(18:3(6Z,9Z,12Z)/P-18:1(11Z))	HMDB0009149	1.66	1.49E-02	1.85	−2.29
791.61409	9.28	PS(O-16:0/21:0)	NA	2.17	1.09E-04	1.94	−3.40
757.56178	13.56	PE-NMe(18:1(9E)/18:1(9E))	NA	1.32	3.37E-02	2.05	1.14
743.54755	17.01	PE(18:1(11Z)/18:1(11Z))	HMDB0009025	1.57	7.66E-03	2.11	−0.48
927.79024	16.59	PE-NMe(24:0/24:1(15Z))	NA	1.90	1.12E-04	2.18	−0.92
829.55974	11.97	PC(18:2(9Z,12Z)/22:6(4Z,7Z,10Z,13Z,16Z,19Z))	HMDB0008156	1.49	1.17E-02	2.24	0.21
717.53171	17.01	PE(16:0/18:1(11Z))	HMDB0008926	1.71	2.37E-03	2.57	−1.41
803.54473	11.96	PC(16:1(9Z)/22:6(4Z,7Z,10Z,13Z,16Z,19Z))	HMDB0008023	1.75	1.22E-03	2.80	−0.52
753.52851	17.00	PE-NMe(18:2(9Z,12Z)/18:2(9Z,12Z))	NA	1.47	1.38E-02	3.79	0.36

### Pathway analysis of differential metabolites

**FIGURE 4:**
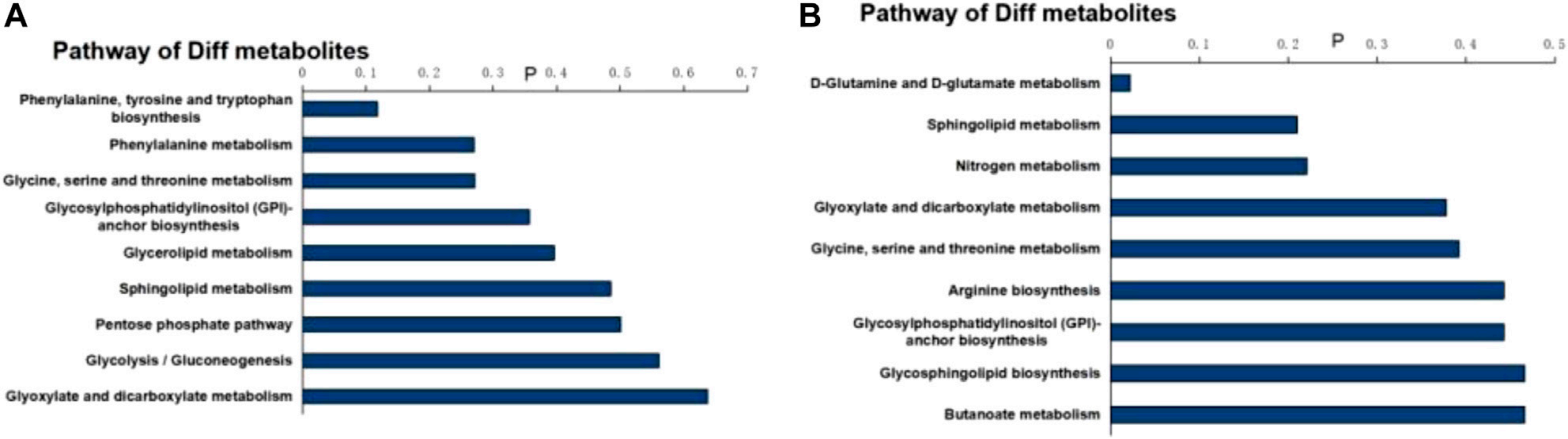
Pathway analysis of differential metabolites. **(A)** Bar chart of the metabolic pathway of NC *versus* LPS. **(B)** Bar chart of the metabolic pathway of LPS + Ber *versus* LPS.

**FIGURE 5:**
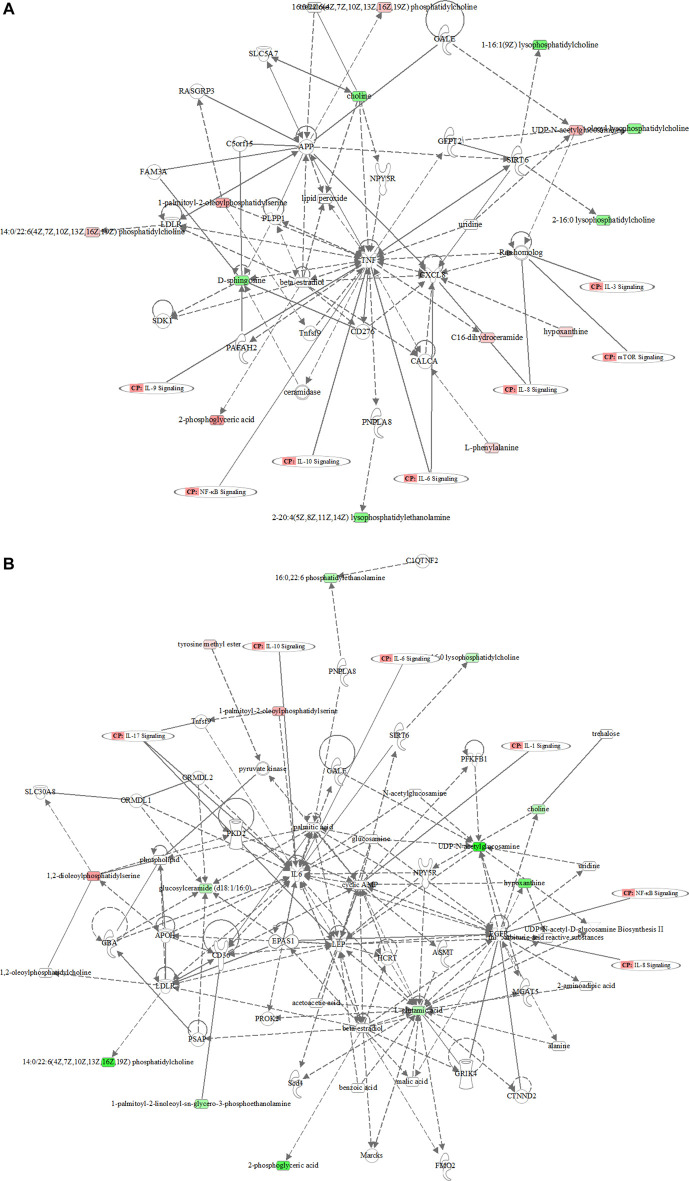
The metabolic network profile. The map was gained by IPA software. **(A)** The network analysis of different metabolites between the LPS and control groups. **(B)** The network analysis of different metabolites between the LPS and LPS + Ber groups.

MetaboAnalyst 3.0 was used for pathway analysis using the metabolites identified in Table 1 between the control, the LPS, and LPS + Ber groups, respectively, as shown in Figures 4A,B. The pathways of phenylalanine, tyrosine and tryptophan biosynthesis, phenylalanine metabolism, glycosylphosphatidylinositol- (GPI-) anchor biosynthesis, and glycolipid metabolism were significantly altered in the cases of NC *versus* LPS group. We also found that the significantly altered pathways mainly involve glycerolipid metabolism in LPS *versus* LPS + Ber group. This pathway is consistent with the pathway induced by LPS, indicating that berberine could regulate the metabolic changes induced by LPS. In addition, the regulated metabolites were imported into the IPA software for network construction, as shown in Figures 5A,B, The differentially expressed metabolites were enriched in pathways associated with apoptosis and inflammatory signaling pathway, including the nuclear factor kappa B (NF-ΚB) signaling pathway, mechanistic target of rapamycin (mTOR) signaling pathway, and IL-6 signaling pathway.

### Effects of berberine on the cell viability of HGFs

The viability of the HGFs was determined by the cell counting kit-8 (CCK8) assay to evaluate the toxic effects of berberine. No toxic impact on HGFs at the doses of 0.1, 0.5, 1, 2.5, 5, 10, 25, 50, and 100 μM was observed. The 5, 10, and 25 μM groups showed relatively increased cell proliferation at 24 and 48 h (*p* < 0.05).

### Effects of berberine on HGFs cell viability and apoptosis

**FIGURE 6:**
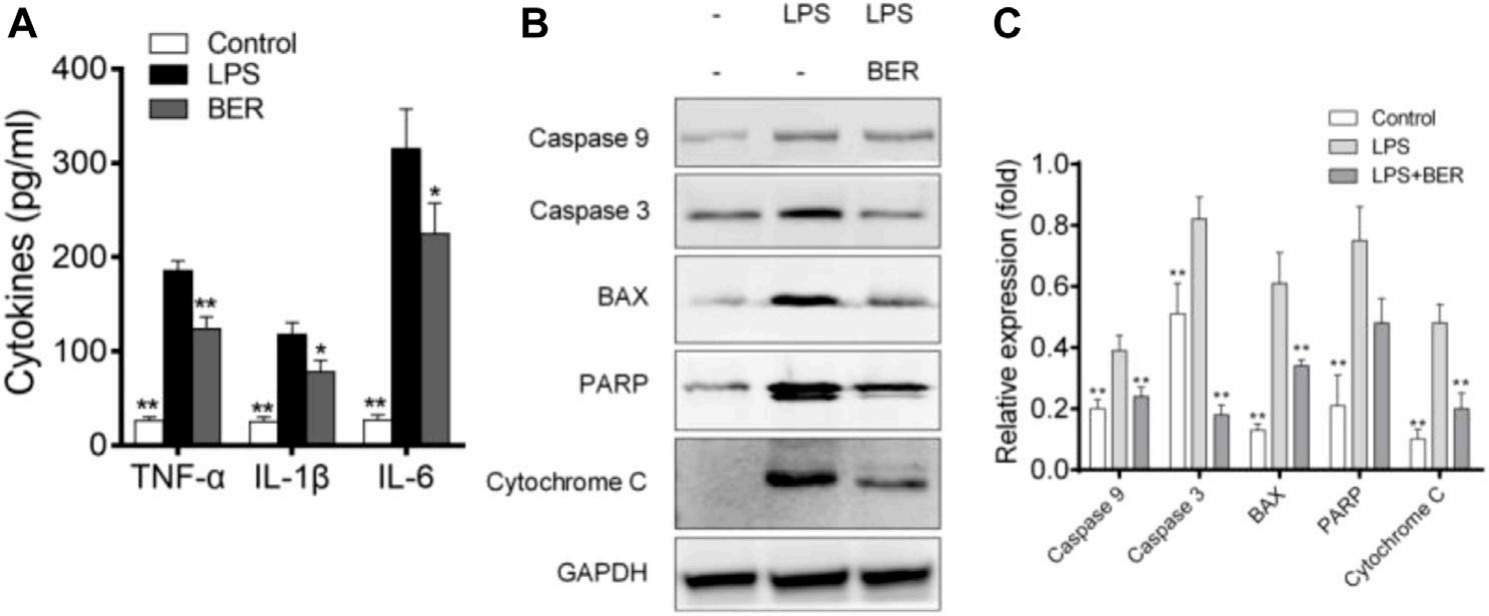
**(A)** Berberine inhibits LPS-induced IL-6, IL-1*β*, and TNF-*α* production in HGFs. The data presented are the means ± SD of three independent experiments. #*p* < 0.05 *versus* the control group; *p < 0.05, **p < 0.01 *versus* the LPS group. **(B)** Effects of berberine on BAX, PARP, Caspase-9, Caspase-3, and cytochrome C expression. The values presented are the means ± SD of three independent experiments. **p < 0.01, LPS *versus* NC group, LPS *versus* LPS + Ber group. **(C)** Effects of berberine on BAX, PARP, Caspase-9, Caspase-3, and cytochrome C expression. The values presented are the means ± SD of three independent experiments. **p < 0.01, LPS *versus* NC group, LPS *versus* LPS + Ber group.

**FIGURE 7:**
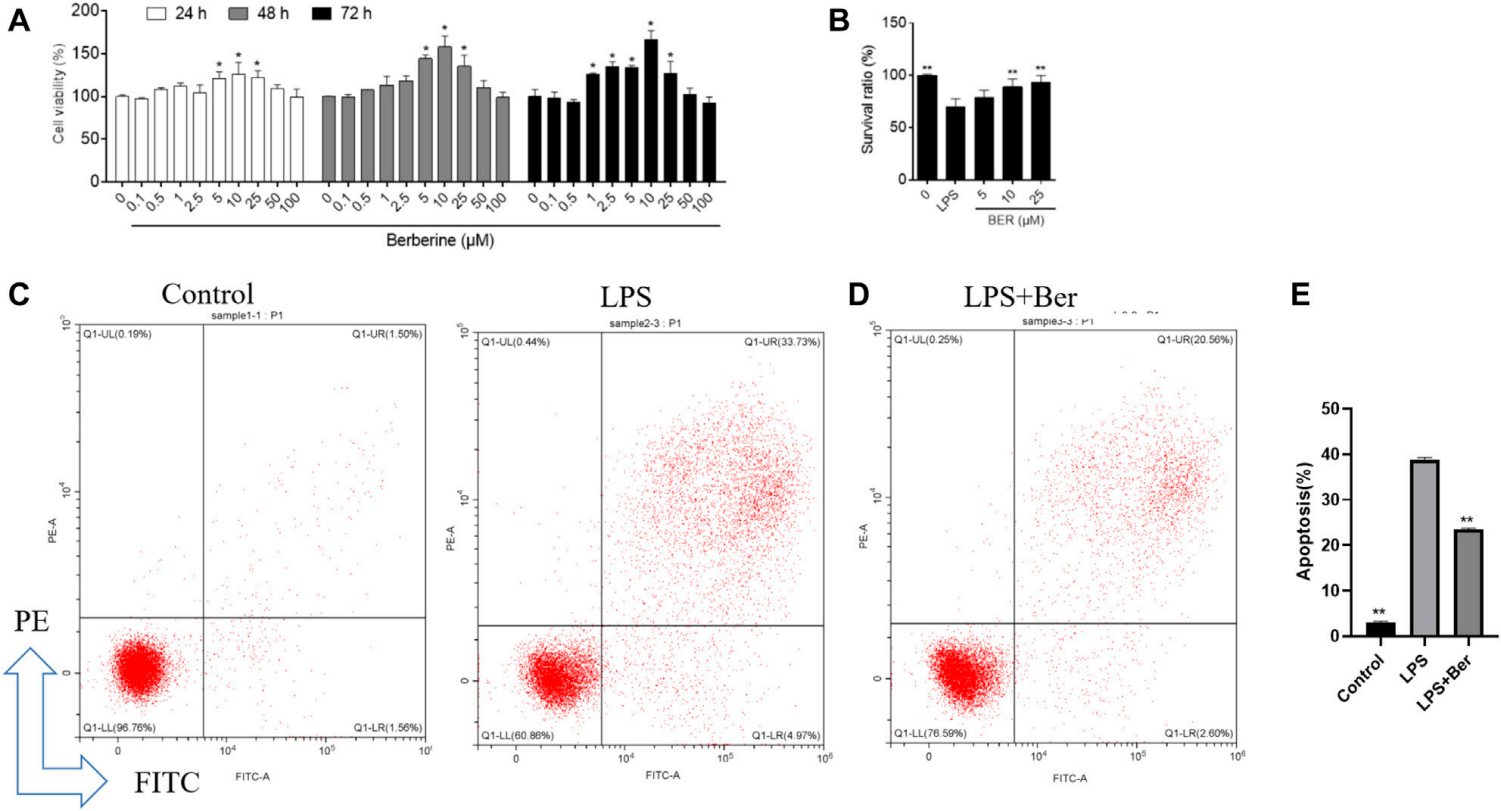
**(A)**, Cellular viability using cell counting kit-8 assay on 24, 48, and 72 h following treatment with different concentrations of berberine. *p < 0.05, compared with the control group on day 5. ^#^ *p* < 0.01. **(B)** Effect of berberine on HGFs viability on 24 h with different concentrations of berberine (5, 10, and 25 μM). **(C,D)** Flow cytometry analysis was conducted to examine the effects of LPS and berberine on apoptosis in HGFs on 24 h (berberine, 10 uM, LPS, 1 μg/ml). Early (annexin-Vpos/PIneg) and late (annexin-Vpos/PIpos) apoptotic cells can be discriminated from vital (Annexin-Vneg/PIneg) or necrotic (annexin-Vneg/PIpos) cells according to their fluorescence emission. **(E)** Dot plots representative of 24 h treated cells are shown. The graph shows the mean cell percentages (±SD, *n* = 3) of viable, early, and late apoptotic and necrotic cells.

Berberine could inhibit the secretion of IL-6, IL-1*β*, and TNF-α induced by LPS in HGF (Figure 6A) and the expression of apoptosis-related proteins, including BAX, PARP, Caspase-9, Caspase-3, and cytochrome C as shown in Figures 6B,C. Cell viability was measured by cell counting kit-8 (CCK8) assay to evaluate the toxic effects of berberine. No toxic impact on HGFs at the doses of 1, 5, 10, 25, 50, and 100 μM (Figures 7A,B) was observed. The 5, 10, and 25 μM groups showed relatively increased cell proliferation at 24 and 48 h (*p* < 0.05). Berberine (10 uM) could inhibit LPS-induced apoptosis in HGFs at 24 h, as shown in Figures 7C–E.

## Discussion

Periodontitis is a chronic disease of the tooth-supporting apparatus caused by specific dental bacteria, resulting in progressive loss of attachment, tooth mobility, and, finally, tooth loss (Liao et al., 2019). Due to the importance of HGFs in maintaining the integrity of periodontal tissue and the regulation of periodontal inflammation, given the anti-inflammatory effect of berberine, it is of great significance to find the biological function of berberine on HGFs (Zhai et al., 2020). In our study, we determine the possible anti-inflammatory and metabolic regulation effects of berberine treatment periodontitis using the LPS-induced HGFs inflammatory model.

Our results showed that some PS, PE, PC, and TG significantly decreased in the LPS group. Besides, our results also observed an increase in some PE-NMe in the LPS group compared with the control group, which suggested the dysfunction of glycerophospholipids metabolism in the LPS group. Glycerophospholipids contain many derivative forms, which are structural components of cell membranes. In our study, these lipids with the most robust downregulation were shown in PS (24:0/24:1(15Z)), TG (20:2/20:4/20:4), TG (20:1/20:1/20:4), PS (O-18:0/19:1), PS (15:0/20:1), and LysoPC (16:1/0:0) in the LPS group *versus* NC group. However, in the LPS + Ber group, these levels were similar to the control group, so berberine showed an obvious reversal effect of LPS-induced metabolic disorder. We also found that PE-NMe (24:0/24:1), PC (18:2/22:6), PE (16:0/18:1), PC (16:1/22:6), and PE-NMe (18:2/18:2) were higher in the LPS group, which might be the important biomarkers for the diagnosis, prognosis, and treatment of periodontitis. Further comparative research is needed. At present, there is no study on these substances as markers of periodontitis (Wishart, 2019).

PS occurs in free form and as part of eukaryotic cell membranes and organelles. They can release fatty acids by phospholipases and other enzymes. These fatty acids could be metabolized to produce several activating lipids that can directly influence inflammation (Feng et al., 2019). PS comprises a minor percentage of a phospholipid, but it is crucial for cell signaling and blood coagulation (Rodrigues et al., 2021). It is well known that PS-mediated apoptotic body recognition is an important phagocytic response (Rodrigues et al., 2021). Macrophages are reprogrammed to secrete proinflammatory mediators, for instance, IL-10 and transforming growth factor-B (Sakanaka et al., 2021). PS-containing liposomes have been used as a strategy to ultimately extinguish inflammation (Yan et al., 2015). Our results showed that berberine contributes beneficial effects in suppressing inflammation by regulating PS, suggesting the potential to attenuate periodontitis.

Phosphatidylcholine (PC) is a major component of membranes and an essential phospholipid in mammalian cells and tissue; 96% of choline in animal tissues exists as PC, which is synthesized *via* the CDP-choline pathway and PE N-methyltransferase pathway (Fischer et al., 2020). Generally, lysophosphatidylcholine (LysoPC) is produced by hydrolysis of PC by phospholipase A2. PC and LysoPC were important signaling molecules correlated with chronic inflammation and tissue damage (Calianese and Birge, 2020). Sphingomyelin (SM) is a kind of sphingophospholipid that serves as a building block for cell membranes and is synthesized by ceramide and phosphorylcholine from PC (Chakrabarti, 2021). The metabolites of SM include ceramide, sphingosine, and sphingosinephosphate. Ceramide is the key metabolite of sphingomyelin metabolism. Its biological functions primarily include inducing apoptosis (Toshima et al., 2019; Partoazar et al., 2021), regulating cell differentiation (Naeini et al., 2021), regulating cellular immunity, and participating in regulating inflammatory response (Aragón-Aranda et al., 2021). Our results revealed that berberine could significantly decrease LysoPC levels, which indicated that berberine could significantly prevent LPS-induced systemic inflammation by regulating the abnormal phospholipid metabolic pathway.

In our study, berberine can regulate not only the abnormal metabolic pathway, but also the critical pathways of inflammatory and apoptosis induced by LPS in HGFs. We found that the inflammatory response during LPS stimulation altered the inflammation microenvironment, and such a change leads to the HGFs being more susceptible to apoptosis. In addition, periodontitis is associated with abnormal lipid metabolism (Liu et al., 2021), and apoptosis of HGFs may be induced by oxidative stress during apoptosis. In the current study, berberine decreased LPS-induced apoptosis in HGFs cells. Subsequently, the apoptosis-associated factors were examined by western blot analysis. The data revealed that LPS markedly enhanced the expression levels of BAX, PARP, Caspase-9, Caspase-3, and cytochrome C, whereas berberine reduced their expression levels. Thus, the data suggested that berberine suppressed the apoptosis of the HGFs by downregulating the apoptosis caspase signaling pathway.

## Conclusion

In our research, we examined berberine’s effects on HGFs and the mechanism underlying the inhibition of HGFs viability in an LPS-model *in vitro* using UPLC-MS techniques. The production of IL-6, IL-1*β*, and TNF-*α* increased significantly in the LPS-treated group. However, berberine suppressed LPS-induced IL-6, IL-1*β*, and TNF-*α* production. We found that berberine as a supplement could reduce inflammation and apoptosis induced by LPS. The result of the metabolomics study suggested phospholipid metabolic pathway disruption in the LPS-induced model. However, berberine could reverse the metabolic pathway by regulating the PE-NMe, PS, PC, LysoPC, and SM levels. These findings indicated the pharmacological mechanisms of berberine on HGFs for the treatment of periodontitis.

## Data Availability

The original contributions presented in the study are included in the article/Supplementary Material. Further inquiries can be directed to the corresponding authors.
